# Study Protocol for a Randomized Controlled Clinical Trial on the Outcome of Surgical Versus Primary Nonsurgical Treatment of Traumatic Thoracolumbar Spine Burst Fractures in Patients Without Neurological Symptoms—A34RCT

**DOI:** 10.1227/neuprac.0000000000000091

**Published:** 2024-04-25

**Authors:** Sonja Häckel, Martin N. Stienen, Benjamin Martens, Valentin Neuhaus, Christoph E. Albers

**Affiliations:** * Department of Orthopaedic Surgery and Traumatology, Inselspital, University Hospital Bern, University of Bern, Bern, Switzerland;; ‡ Graduate School for Health Sciences, University of Bern, Bern, Switzerland;; § Department of Neurosurgery, Kantonsspital St. Gallen & Medical School of St. Gallen, St. Gallen, Switzerland;; ‖ Spine Center of Eastern Switzerland, Kantonsspital St. Gallen & Medical School of St. Gallen, St. Gallen, Switzerland;; ¶ Department of Orthopaedic Surgery and Traumatology, Kantonsspital St. Gallen & Medical School of St. Gallen, St. Gallen, Switzerland;; # Division of Trauma Surgery, University Hospital Zurich (USZ), University of Zurich (UZH), Zurich, Switzerland

**Keywords:** Traumatic thoracolumbar burst fractures, Anteroposterior stabilization (360°), Conservative treatment, Randomized controlled trial

## Abstract

**BACKGROUND AND OBJECTIVES:**

There are still major global differences in the treatment of acute traumatic thoracolumbar burst fractures in patients without neurological deficits and without posterior column injury. Treatment strategies range from conservative treatment with orthosis or early functional mobilization to various surgical stabilization techniques. The study's objectives are to evaluate the clinical (Oswestry Disability Index [ODI]) and radiographical outcomes (restoration and maintenance of spinal alignment; injury of the affected intervertebral disk) and determine the prevalence of complications until 24 months of follow-up.

**METHODS:**

The study is designed as a randomized, controlled, noninferiority clinical trial. All patients with a thoracolumbar burst fracture (*Arbeitsgemeinschaft für Osteosynthesefragen* spine type A3 or A4) age 18 to 70 years without neurological deficit and without posterior ligament injury can be enrolled. We will randomly assign 52 patients for either surgical or nonsurgical treatment. The surgical group will receive combined anterior–posterior (360°) spinal stabilization therapy. Participants in the nonsurgical group will be treated with a 3-point hyperextension orthosis for 6 weeks. The primary outcome is the difference in ODI at 2 years after injury.

**EXPECTED OUTCOMES:**

We expect to find that conservative treatment of burst fractures in the thoracolumbar spine will be noninferior to the surgical treatment.

**DISCUSSION:**

This study will provide high-quality data comparing a modern surgical treatment regime with a standardized conservative treatment in patients with thoracolumbar burst fractures.

**ABBREVIATIONS:**
AO: *Arbeitsgemeinschaft für Osteosynthesefragen*
EDC: electronic data capturing
ODI: Oswestry Disability Index.

## GENERAL INFORMATION

Protocol Title: Study protocol for a randomized controlled clinical trial on the outcome of surgical vs primary nonsurgical treatment of traumatic thoracolumbar spine burst fractures in patients without neurological symptoms—A34RCT.

Registry: This study is registered in ClinicalTrials.gov (NCT05769114).

Study Dates: March 2023 to present.

Sponsor/Funding Agency: This study is supported by the Swiss Academy of Medical Sciences (SAMS), protected research time: 75.000 CHF, Swiss Orthopaedics Grant: 20.000 CHF, OrthoTeam Bern: 10.000 CHF, SUVA: 105.000 CHF, and Bangerter-Rhyner Stiftung: 60.000 CHF. The funders did not have a role in the design or conduct of the study and the authors have ultimate authority over study activities. The content is solely the responsibility of the authors.

Institutional Approvals: Institutional Review Board (Cantonal Ethic Committees Bern, Switzerland [Project ID 2022-00662]).

Roles and Responsibilities: SH and CEA conceived the study, organized the funding, and conducted the study at the main site in Bern. MNS is the principal investigator at the site in St. Gallen. BM contributes as a study physician at the site in St. Gallen.

## RATIONALE AND BACKGROUND INFORMATION

**FIGURE 1.**
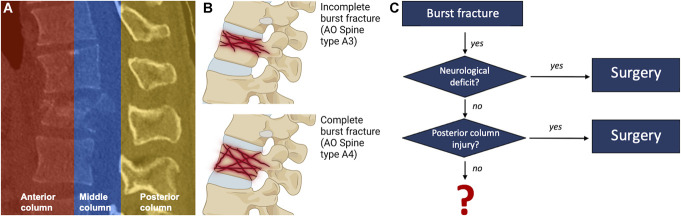
**A**, Biomechanical three-column model of the spine published by Denis in 1983. Compression forces on the anterior column result in vertebral body fracture, whereas tension forces on the posterior column led to a ligamentous injury. **B**, Example of a complete (one endplate of the vertebral body injured) vs an incomplete vertebral body fracture (both endplates of the vertebral body fractures). **C**, Decision-making tree for treatment of burst fractures: neurological deficits and posterior column injury are clear indications for surgical treatment. On the contrary, the optimal treatment for neurologically intact patients without a posterior column injury is still unclear. *Created with* BioRender.com. AO, *Arbeitsgemeinschaft für Osteosynthesefragen*.

Most spinal fractures occur at the thoracolumbar transition zone, which includes the 10th thoracic to the 3rd lumbar vertebra.^1,2^ Spinal fractures are classified according to the *Arbeitsgemeinschaft für Osteosynthesefragen* (AO)-Spine classification of thoracolumbar fractures. This classification system separates 3 major types (A, B, and C) with increasing injury severity based on the involvement of the functional columns (anterior, middle, and posterior), mechanism of injury, and fracture morphology (Figure 1A). Incomplete (AO type A3) and complete burst fractures (AO type A4) are distinguished by the involvement of one or both endplates of the vertebral body (Figure 1B). A high consensus exists that traumatic burst fractures with neurologic deficit, as well as those with disruption of the posterior tension band complex (posterior column), experience better outcomes with surgical treatment compared with nonsurgical (Figure 1C).^3^ However, no treatment recommendation exists for traumatic burst fractures without a neurologic deficit and without the involvement of the posterior column.^3,4^

We hypothesize that nonsurgical management of thoracolumbar burst fractures will be noninferior to surgical treatment in patients without neurological deficits and without injury of the posterior column.

## STUDY GOALS AND OBJECTIVES

### Primary Goals

To investigate whether nonsurgical treatment with bracing (possibly followed by surgery if there is a deterioration or lack of satisfaction) of an acute traumatic burst fracture of the thoracolumbar region (10th thoracic to 3rd lumbar vertebral body) without injury of the posterior tension band/posterior column and neurological deficits is noninferior to the surgical treatment concerning the clinical outcome.

### Secondary Goals

The secondary objectives of the study are to investigate

1. The changes in spinal alignment/kyphosis, the grade of intervertebral disk degeneration, and healing rate (nonsurgical group) or spinal fusion rate (surgical group),
2. The patient-reported outcomes of the surgical and nonsurgical treatment groups,
3. The costs comparing surgical vs nonsurgical treatment options,
4. Patients' outcomes on incomplete vs complete burst fractures.

## STUDY DESIGN

This study is designed as an investigator-initiated, multicenter randomized controlled clinical trial. The study protocol complies with the SPIRIT 2013 statement. The selection criteria are presented in Table.

**TABLE.** Selection Criteria

Inclusion criteria	Exclusion criteria
Acute traumatic burst fracture of the thoracolumbar spine (10th thoracic to 3rd lumbar vertebral body)	Injury of the posterior tension band/posterior column of the thoracolumbar spine^5,6^
Age 18-70 y at inclusion	Any neurological deficit (American Spinal Injury Association Impairment Scale Grade A-D)^7^
Informed consent for study participation	Pathological vertebral body fractures (suspected by MRI, computed tomography scan, and patient history)^a^
	Concomitant spinal fractures at any other level of the spine outside the T10-L3 level^a^
	Multiple trauma or Injury Severity Score ≥16 or additional injuries^a^
	Any known previous spinal surgery in the thoracolumbar spine^a^
	Any severe, progressive, or uncontrolled medical or psychiatric condition^a^
	Known history of substance abuse (ie, recreational drugs, alcohol)^a^
	Any severe systemic medical disease that would exclude the patient from being a potential candidate for surgery
	Pregnancy or women planning to conceive within the study period. All women included in this study must have a negative blood pregnancy test (human chorionic gonadotropin blood level at visit 1. If pregnancy occurs during the study period, the patients drop out of the study)
	Inability to follow the procedures of the study, eg, because of inability to understand German, French, or English^a^

a Which, in the opinion of the research/investigative team, would compromise (or interfere with) patients' ability to participate in the study.

## METHODOLOGY

### Study Setting

**FIGURE 2.**
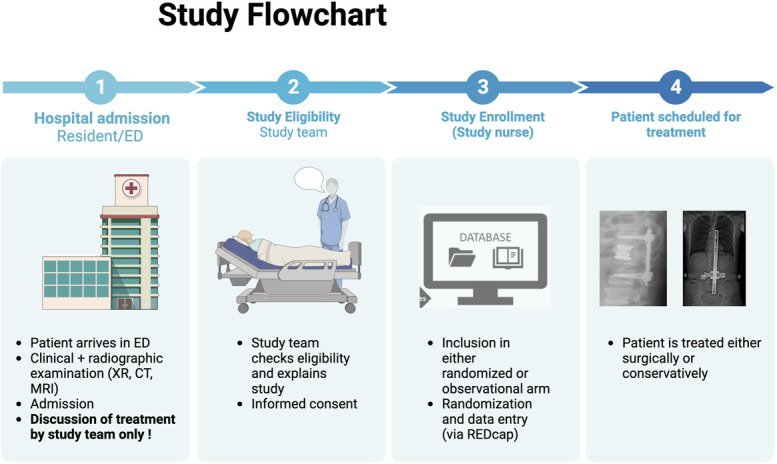
Study flowchart. CT, computer tomography; ED, emergency department; REDCap, research electronic data capture; XR, x-ray. Created with Biorender.com.

The study is conducted in Switzerland. The study site is the Inselspital, University Hospital Bern, which is a level 1 trauma center. The other sites are the Spine Center of Eastern Switzerland, Cantonal Hospital St. Gallen, and Department of Traumatology, University Hospital Zurich, Zurich, Switzerland. The addition of more study sites will depend on the enrollment status. The selection criteria are presented in Table. Flowchart is shown in Figure 2.

### Interventions

#### Surgical Treatment Protocol: Anterior–Posterior Stabilization With an Expandable Cage

**FIGURE 3.**
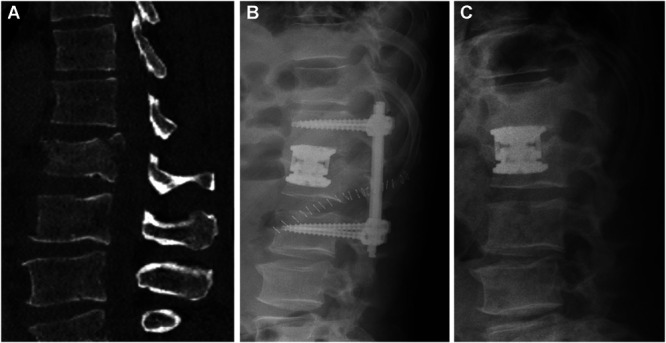
Case example of a surgically treated (incomplete, *Arbeitsgemeinschaft für Osteosynthesefragen* spine type A3) burst fracture of the 3rd lumbar vertebrae in a 39-year-old man. **A**, Initial computed tomography scan showing the fractured vertebrae involving the upper endplate. **B**, Postoperative x-ray after implantation of an expandable cage. **C**, X-ray after dorsal implant removal showing the preserved spinal alignment.

Surgical treatment will be performed under general anesthesia. With the patient in a prone position, the surgeon will perform a minimally invasive (percutaneous) posterior stabilization of the injured spinal segments (Figure 3A and 3B). In a second stage procedure performed on the same day or separately, a (partial) corpectomy will be performed (Figure 3C). Immediately after surgery, the patient can start mobilization. After the hospitalization, weekly physiotherapy visits are prescribed. In the first 6 weeks, the rehabilitation program includes isometric training, activation, and circulation promotion. After 6 weeks, range-of-motion exercises can be added. Spinal fusion will be confirmed by a computed tomography scan 6 months after the index procedure before the removal of the posterior implants (pedicle screw/rod system).

#### Nonsurgical Treatment Protocol

All patients will receive external bracing (3-point hyperextension brace) for 6 weeks, which must always be worn except when lying flat in bed. After hospital admission, bed rest is prescribed until an experienced orthopedist adjusts the brace. Also, a physiotherapist will instruct the patient to accomplish daily activities with the restriction he or she will have by wearing the brace. Discharge is possible if the patient feels comfortable with the brace and mobilization as well as if adequate pain management has been achieved. After discharge, weekly physiotherapy sessions commence in the week after injury. Initially, the rehab program encompasses isometric exercises, activation routines, and methods to enhance circulation for the first 6 weeks. Subsequently, exercises aimed at improving range of motion are introduced after this period.

## DISCUSSION

To date, only 2 randomized controlled trials have been published comparing surgical vs nonsurgical treatment for traumatic burst fractures.^5,8^ In the study by Wood et al, superior clinical, functional, and equivalent radiographic outcomes were evident after nonsurgical treatment. By contrast, the study by Siebenga et al reported improved clinical, functional, and radiographic results with surgical treatment.^5,8,9^ Particularly, the study by Wood et al, favoring conservative treatment, was limited by a lack of standardization of the treatment protocols. In the surgical group, different and nowadays obsolete surgical techniques and implants were used. Nonsurgical treatment consisted of body casting for up to 12 weeks and additional bracing for another 4 to 8 weeks, resulting in an overall treatment duration of almost half a year.

In the realm of burst fracture treatment, AOSpine spearheaded a prospective multicenter clinical trial focused on this issue.^10^ Moreover, the StUdy oN Burst Fractures (SUNBURST),^11^ a current randomized controlled, registry-based trial, is underway. However, this trial is characterized by variability in surgical treatment across different centers.

To our knowledge, this study is pioneering in comparing nonsurgical with surgical treatment modalities incorporating a standardized 360° stabilization technique for the spine.

## TRIAL STATUS

Enrollment for this study is currently ongoing.

## SAFETY CONSIDERATIONS

Safety assessments will be conducted through physical examinations, analysis of radiographic imaging, and the documentation of any adverse events, including serious adverse events. If there will be a clinical indication (spinal instability measured by a kyphosis >30° on x-ray,^12,13^ intolerable pain, and delayed onset of neurological symptoms) or if the patient is not satisfied after treatment, surgery (anteroposterior stabilization) will be recommended.

### Harms

A causality assessment of the event to the trial intervention (based on the terms given in the International Council on Harmonisation-E2A with modifications) will be done. Any event assessed as possibly, probably, or definitely related is classified as related to the trial intervention. We will make a severity assessment of the event according to the terminology of the Common Terminology Criteria for Adverse Events, version 5.0.^6^ All serious adverse events are documented and reported immediately (within a maximum of 24 hours) to the sponsor-investigator.

## FOLLOW-UP

Patients will be seen on arrival at the emergency department/outpatient clinic to obtain relevant medical history and adverse event collection. After obtaining signed informed consent, patients will be randomly assigned to 1 of the 2 treatment arms. If randomization is refused, the patient will be asked to participate in the observational arm, which is then treated as the current standard operating procedure at our hospital (surgical treatment). After discharge, conservatively treated patients will be seen for an additional visit in the outpatient clinic for 2 weeks (±5 days). Afterward, all participants will have routine follow-up at 6 weeks (±7 days), 13 weeks (±14 days), and 26 weeks (±30 days) after discharge to obtain radiographic images, clinical examination, and adverse event collection. Surgical-treated patients will undergo removal of the posterior instrumentation after 6 to 9 months. Patients will then be seen for follow-up after 1 (±30 days) and 2 years (±45 days) (**Supplemental Digital Content 1**, http://links.lww.com/NS9/A14).

## DATA MANAGEMENT AND STATISTICAL ANALYSIS

Data collection will be performed electronically using a dedicated electronic data capturing (EDC) system (Research Electronic Data Capture). Responsibility for hosting the EDC system and the database lies with CTU Bern.

The primary end point will be Oswestry Disability Index (ODI) change from baseline at 104 weeks. The report assumes that baseline ODI will be 45 and 1-year ODI will be 25 (a difference of 20), as we assume noninferiority, the expected mean difference in both groups is the same.^7,8,14^ Assuming that the difference between baseline and 1-year follow-up is 20 ODI points, the SD of the difference is 6 (based on previously collected data of a prospective trial^15^), and the noninferiority margin is 5.5, 38 participants in total (19 in each arm) would be needed to show noninferiority. By assuming a dropout rate of 20% and a possible crossover from the nonsurgical to the surgical group, we will enroll a total of 52 patients (26 in each arm).

## QUALITY ASSURANCE

Training in the study protocol and management algorithms has been provided to site investigators and their surgical teams. Oversight of the study will be managed directly by the CTU Bern, which includes the verification of source data. To maintain high standards of quality, the study will conduct training sessions for all investigators to promote uniform data collection. Regular visits to monitor the study will also be carried out to ensure the accuracy of the data recorded in the case report forms.

## EXPECTED OUTCOMES OF THE STUDY

We anticipate that nonsurgical treatment of thoracolumbar burst fractures will demonstrate noninferiority compared with surgical intervention in patients who do not present with a neurological deficit and where there is no injury to the posterior column.

## DURATION OF THE PROJECT

The planned study duration from the first participant's first visit to the last participant's last visit is 4 years. The planned recruitment period from first participant's first visit to the last participant's first visit is 2 years. The study duration for each participant is 2 years.

## PROJECT MANAGEMENT

Principle Investigator: Christoph E. Albers, MD.

Co-Investigators: Sonja Häckel, MD; Martin Stienen, MD.

Statistician: CTU Bern.

## ETHICS

This study is conducted in compliance with the protocol, the current version of the Declaration of Helsinki,^16^ International Council on Harmonisation-E6 (Good Clinical Practice),^17^ the Swiss Human Research Act,^18^ as well as other locally relevant legal and regulatory requirements. The study protocol (Version 6.1, 14.12.2023) was reviewed and accepted by the ethics committee at the Cantonal Ethic Committees (Project ID 2022-00662). Protocol amendments: necessary changes to the study protocol (amendments) will be presented to the ethics committees for review. The study was registered before initiation at clinicaltrials.gov (NCT05769114) and the Swiss National Clinical Trial Platform.

### Ethics and Dissemination

Study results will be reported no matter what the outcomes are in a reputable peer-reviewed scientific journal and according to the Consolidated Standards of Reporting Trials statement. The author sequence will be discussed transparently depending on participation and contribution (recruitment numbers) to the study. The results will be published for open access.

### Protocol Amendments

The institutional review board will review and approve any amendments to the study protocol as well as the site-specific informed consent documents. Documentation related to the study will undergo scrutiny by the ethics review board as part of the approval process.

### Consent and Assent

Participants must provide formal consent using the designated approval form before undergoing any procedures specific to the study. It is imperative that patients fully comprehend the nature of the research study, its aims, as well as the potential risks and benefits involved in participating. A minimum period of 24 hours will be provided to allow for any inquiries and for the participant to make an informed decision regarding their involvement.

### Confidentiality

The investigator will protect the privacy of the participants, ensuring they are not named in any documents outside the study site. Participants’ confidentiality is maintained through the use of ID codes. Only the study team has access to the detailed data, including the ID list, for purposes such as monitoring and auditing. The EDC system and its database are securely stored in a locked room, with access limited to system administrators. A role-based access system, secured with personal passwords, controls each user’s access to the system and database according to their specific roles.

### Access to Data

All study investigators maintain the right to data access.

## SUPPLEMENTAL DIGITAL CONTENT

**Supplemental Digital Content 1**. **Table 1**. Visit schedule (randomized- and observational arm).

## References

[1] Singh K Saxena A Vaccaro AR. Thoracic and lumbar spine trauma. In: Vaccaro AR, ed. Core Knowledge in Orthopaedics: Spine. Elsevier; 2005:290-304.

[2] Leucht P Fischer K Muhr G Mueller EJ. Epidemiology of traumatic spine fractures. Injury. 2009;40(2):166-172. 19233356 10.1016/j.injury.2008.06.040

[3] Thoracic and lumbar trauma. Accessed January 22, 2022. https://surgeryreference.aofoundation.org/spine/trauma/thoracolumbar

[4] Spiegl UJ Josten C Devitt BM Heyde CE. Incomplete burst fractures of the thoracolumbar spine: a review of literature. Eur Spine J. 2017;26(12):3187-3198. 28547575 10.1007/s00586-017-5126-3

[5] Siebenga J Leferink VJM Segers MJM Treatment of traumatic thoracolumbar spine fractures: a multicenter prospective randomized study of operative versus nonsurgical treatment. Spine (Phila Pa 1976). 2006;31(25):2881-2890. 17139218 10.1097/01.brs.0000247804.91869.1e

[6] National Cancer Institute. Common terminology criteria for adverse events (CTCAE) v5.0; 2017. Accessed January 22, 2022. https://www.meddra.org/

[7] Kumar A Aujla R Lee C. The management of thoracolumbar burst fractures: a prospective study between conservative management, traditional open spinal surgery and minimally interventional spinal surgery. Springerplus. 2015;4(1):204. 25969819 10.1186/s40064-015-0960-4 PMC4418977

[8] Wood K Butterman G Mehbod A Garvey T Jhanjee R Sechriest V. Operative compared with nonoperative treatment of a thoracolumbar burst fracture without neurological deficit: a prospective, randomized study. J Bone Joint Surg Am. 2003;85(5):773-781. 12728024 10.2106/00004623-200305000-00001

[9] Wood KB Buttermann GR Phukan R Operative compared with nonoperative treatment of a thoracolumbar burst fracture without neurological deficit. J Bone Joint Surg. 2015;97(1):3-9. 25568388 10.2106/JBJS.N.00226

[10] Thoracolumbar burst fractures study comparing surgical versus non-surgical treatment. ClinicalTrials.gov. Accessed January 27, 2024. https://clinicaltrials.gov/study/NCT02827214

[11] Blixt S Mukka S Försth P Westin O Gerdhem P. Study protocol: the SunBurst trial—a register-based, randomized controlled trial on thoracolumbar burst fractures. Acta Orthop. 2022;93:256-263. 35175357 10.2340/17453674.2022.1614

[12] Öner FC Wood KB Smith JS Shaffrey CI. Therapeutic decision making in thoracolumbar spine trauma. Spine (Phila Pa 1976). 2010;35(21 Suppl):S235-S244. 20881467 10.1097/BRS.0b013e3181f32734

[13] Cahueque M Cobar A Zuñiga C Caldera G. Management of burst fractures in the thoracolumbar spine. J Orthop. 2016;13(4):278. 27408503 10.1016/j.jor.2016.06.007 PMC4930335

[14] Azhari S Azimi P Shahzadi S Mohammadi HR Khayat Kashani HR. Decision-making process in patients with thoracolumbar and lumbar burst fractures with thoracolumbar injury severity and classification score less than four. Asian Spine J. 2016;10(1):136-142. 26949469 10.4184/asj.2016.10.1.136 PMC4764525

[15] Thoracolumbar burst fractures study comparing surgical versus non-surgical treatment - full text view. ClinicalTrials.gov. Accessed March 4, 2022. https://clinicaltrials.gov/ct2/show/NCT02827214

[16] WMA Declaration of Helsinki – ethical principles for medical research involving human subjects – WMA – The World Medical Association. Accessed January 22, 2022. https://www.wma.net/policies-post/wma-declaration-of-helsinki-ethical-principles-for-medical-research-involving-human-subjects/

[17] International Council for Harmonisation of Technical Requirements for Pharmaceuticals for Human Use (ICH). ICH Harmonised Guideline Integrated Addendum to ICH E6(R1): Guideline for Good Clinical Practice E6(R2); ICH: 2016.

[18] SR 810.30 - Bundesgesetz vom 30. September 2011 über die Forschung am Menschen (Humanforschungsgesetz, HFG). Accessed January 22, 2022. https://www.fedlex.admin.ch/eli/cc/2013/617/de

